# Sensory-motor and cardiorespiratory sensory rehabilitation associated with transcranial photobiomodulation in patients with central nervous system injury: Trial protocol for a single-center, randomized, double-blind, and controlled clinical trial

**DOI:** 10.1097/MD.0000000000015851

**Published:** 2019-06-21

**Authors:** Ana Paula Pinto, Carolina Lobo Guimarães, Gabriela Aparecida da Silveira Souza, Patrícia Sardinha Leonardo, Marcele Florêncio das Neves, Fernanda Pupio Silva Lima, Mário Oliveira Lima, Rodrigo Alvaro Brandão Lopes-Martins

**Affiliations:** a Laboratório de Engenharia de Reabilitação Sensório Motora; b Laboratório de Biofotônica e Terapêutica Experimental, Instituto de Pesquisa e Desenvolvimento, Universidade do Vale do Paraíba, São José dos Campos, São Paulo, Brazil.

**Keywords:** cardiorespiratory rehabilitation, low-level laser therapy, neurological diseases, PBM therapy, photobiomodulation, sensory motor rehabilitation

## Abstract

**Background:**

Central nervous system diseases such as stroke, spinal cord injury, traumatic brain injury, and multiple sclerosis can be fatal or cause sequelae, affecting sensorimotor and cardiorespiratory systems and quality of life. These subjects present a low response to aerobic and resistance exercise, due to decreased recruitment of muscle fibers and reduction of metabolic capacity. Aerobic exercises bring benefits in terms of fatigue retardation, gait improvement, regulation of the autonomic nervous system, neuroprotection of the brain, stimulation of the production of endogenous neutrotransmitters related to general well-being, and a favoring of neuroplasticity. Photobiomodulation (PBM Therapy) (previously known as low-level laser therapy), and especially transcranial PBM Therapy, has shown benefits in animals and humans such as cognitive improvement, memory, and behavioral improvement, including attenuation of depression and anxiety, and increased cortical oxygenation. The aims of this trial will be to evaluate the parameters related to the function of the musculoskeletal and cardiorespiratory system and the impact of PBM therapy on these parameters, as part of a rehabilitation and training program for people with reduced mobility.

**Methods:**

This is a randomized, double-blind, placebo-controlled trial with 3 groups: Control, only cardiorespiratory rehabilitation (CCR), CCR with PBM Therapy (CR-PBM), CCR and placebo PBM Therapy (CR-PlaceboPBM). n = 90, 30 per group. PBM Therapy parameters: 810 nm laser, 0.028 cm^2^, 100 mW, 3.5 W/cm^2^, 30 seconds per point, 3 J per point, 107.1 J /cm^2^ to 3 electroencephalogram points F7 and F8 and AFz. The trial will be conducted at the University Clinics and the sessions will be 1 hour twice a week for 9 weeks. Baseline, intermediate (4th week), final (9th week), and 2-month follow-up will be performed. Muscular activation, heart rate variability, lung volumes and capacities, fatigability, exercise tolerance, cognition, and quality of life at baseline will be evaluated. Subsequent to baseline evaluations, the PBM Therapy groups will be offered laser therapy (active or inactive); all groups will then receive CCR.

**Trial registration number:**

*ClinicalTrials.gov* ID - NCT03751306 (approval date: November 22, 2018).

## Abstract 2

Supplemental Digital Content is available in the text

## 1 Introduction

Neurological diseases account for 16.8% of global deaths and about 7% of disability-adjusted life years (DALYs). Cerebral pathologies are the second cause of death worldwide.^[1]^ Neurological diseases include those of the central nervous system, such as stroke, spinal cord injury, cranioencephalic trauma, and multiple sclerosis. All these pathologies generate some level of dysfunction, which negatively affects quality of life and also incurs high medical costs.^[2–5]^

Spasticity is an incapacitating neurological disorder that can have as etiology the above-mentioned diseases among others of the upper motor neurons.^[6–8]^ Muscular dysfunctions related to spasticity are hyperactivity and hypertonicity, muscle weakness, and contractures.^[8]^ There is also an increase in energy expenditure for performance of dynamic movements, increase in the production of lactic acid and the use of muscle glycogen, and decrease in the synthesis of fatty acids.^[9]^

In addition to muscle changes, spasticity also alters cardiorespiratory and metabolic function. There is evidence of a reduction of peripheral vessel diameter in an experimental model,^[10]^ arrhythmia symptoms, increased sympathetic stimulation to the heart and of high medullary lesions, and decreased cardiac contractility.^[11]^

In the systematic review by Block et al,^[12]^ it was demonstrated by the remote monitoring method that patients with neurological sequelae are not able to perform the recommended exercise time, and have a decreased cardiorespiratory fitness in relation to the population that does not have neurological dysfunctions. This is due to the pathophysiology of the disease-altering physical inactivity through prolonged sedentary behavior.

It is well understood that aerobic physical training improves cardiorespiratory fitness in the general healthy population and also in several disease groups. The application of aerobic training in individuals with central nervous system diseases is increasing due to the benefits demonstrated in the literature, such as improvement of cognition, cardiovascular conditioning, reduction of anxiety and depression symptoms, and increased oxygen consumption efficiency.^[13–16]^

Significant evidence demonstrates that PBM Therapy directly affects mitochondrial functioning, increases microcirculation, reduces pain, decreases inflammatory processes, reduces muscle fatigue, and facilitates improvement in scarring processes.^[17,18]^

PBM Therapy is considered a noninvasive brain stimulation technique, with potential effects on brain plasticity. Phototherapy has been extensively studied and has been shown to be efficient for a variety of clinical procedures.^[19,20]^

PBM Therapy directly influences the activity of cytochrome c oxidase, an electron transport chain enzyme located on the internal membrane of the mitochondria, responsible for the uptake and absorption of light photons within the red and infrared spectra.^[21,22]^

According to Hennessy and Hamblin,^[23]^ if we want to stimulate the brain, we must radiate the laser light directly to the head, more precisely to the forehead, because there is no hair present, which can dissipate/disperse the photons of light. Radiating laser light directly to the forehead would stimulate the frontal lobes of the cortex, the area responsible for motor function, problem solving, memory, language, initiation, and impulse control.

Clinical studies in humans have demonstrated improvements in a wide range of brain functioning, such as cognitive and memory improvement, improvement of the behavioral picture, such as attenuation of depression and anxiety, and increased cortical oxygenation.^[17,24]^

We believe that the same stimulation of brain tissue, that is, the exposure of nervous tissue to PBM Therapy, could also trigger positive effects peripherally. It is proposed here that cardiorespiratory rehabilitation associated with PBM Therapy may increase performance in physical activity in people with reduced mobility.

Therefore, the aims of this trial will be to evaluate the parameters related to the function of the musculoskeletal and cardiorespiratory system and the impact of PBM Therapy on these parameters, through a rehabilitation and training program for people with reduced mobility.

## 2 Methods

### 2.1 Trial design

**Figure 1:**
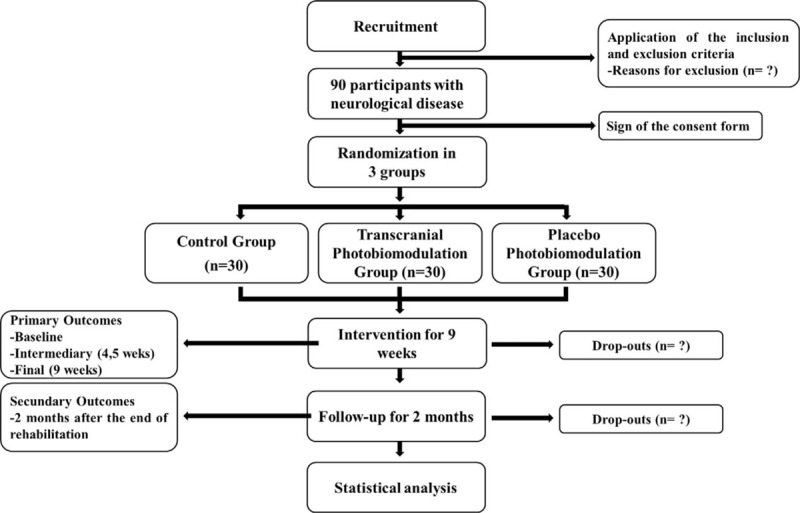
Flow diagram of trial.

It is a unicentric, randomized, double-blind, placebo-controlled trial with 3 groups: control – only cardiorespiratory rehabilitation (CCR), cardiorespiratory rehabilitation with PBM Therapy (CR-PBM), cardiorespiratory rehabilitation and placebo PBM Therapy (CR-PlaceboPBM). The magnitude of the PBM Therapy effect will be compared statistically across groups. In total, there will be 90 patients (30 in each group). The trial will be conducted at the Clinic School of the University of Paraíba (Brazil), and the sessions will be 1 hour, twice a week for 9 weeks. Baseline, intermediate (4th week), final (9th week), and 2-month follow-up will be performed. Further details on the flowchart of the trial design are shown in Fig. 1.

### 2.2 Ethics

The protocol was approved by the Ethics and Research Committee of the University of Vale do Paraíba, Brazil (CAAE 94858718.3.0000.5503), and later registered in ClinicalTrials.gov (NCT03751306). All procedures will be in accordance with the WMA Declaration of Helsinki.

### 2.3 Patients

Patients with reduced mobility due to acquired central nervous system neurological diseases and people with multiple sclerosis were included.

#### 2.3.1 Inclusion criteria

Inclusion criteria are as follows:

(1) People with stroke, traumatic brain injury, spinal cord injury, brain tumor postoperative period, chronic nonprogressive encephalopathy, and multiple sclerosis;
(2) Chronic neurological diseases, from 6 months of injury;
(3) Caucasian individuals;
(4) Age between 18 and 85 years;
(5) Both sexes;
(6) Individuals with cognition preserved;
(7) Persons who are able to wander on the treadmill voluntarily or through assistance of the BrainMov Rehabilitation and Physical Activity Station;
(8) Persons who make continuous and regular use of medications prescribed by the physician for the control and/or treatment of chronic diseases;
(9) Persons with the release of the cardiologist for rehabilitation.

#### 2.3.2 Exclusion criteria

Exclusion criteria are as follows:

(1) Persons who do not meet the inclusion criteria;
(2) Active smokers;
(3) Carriers of chronic respiratory diseases, such as chronic obstructive pulmonary disease, asthma and bronchiectasis;
(4) patients with decompensated heart disease;
(5) Obesity grade II - body mass index greater than 34.99 kg/m^2^;
(6) Patients with spinal cord injury above sixth thoracic vertebra, who present with autonomic dysreflexia;
(7) Patients with American Spinal Injury Association Impairment Scale A or B Impairment;
(8) Patients with multiple sclerosis who are in the onset period;
(9) Patients who use beta-blocking drugs;
(10) Hemorrhagic encephalic vascular accident.

### 2.4 Groups e sample size

The present trial will include 90 patients who will be divided randomly into 3 experimental groups:

(1) Control group (CCR): (n = 30) These individuals will receive only cardiorespiratory rehabilitation; therefore, no PBM Therapy will be applied, either real or placebo.
(2) Transcranial Photobiomodulation Group (CR-PBM): (n = 30) Individuals in this group will receive PBM Therapy followed by cardiorespiratory rehabilitation.
(3) Placebo Group Photobiomodulation (CR-PlaceboPBM): (n = 30) Individuals in this group will receive placebo PBM Therapy; the device will not deliver therapeutic light. Subsequently, they will receive cardiorespiratory rehabilitation.

All patients, after completing the trial, may receive PBM Therapy treatment. Thus, if there is any interest of a patient who did not receive the PBM Therapy during the trial, this will be provided after trial completion

### 2.5 Randomization and blinding

The patients will be recruited from the waiting list of patients of the school clinic and medical records of the institution's former patients. Patients in all 3 groups, CCR (n = 30), CR-PBM (n = 30), and CR-PlaceboPBM (n = 30), must sign consent themselves and must have cognition preserved to participate in the trial. To see Inform consent check supplemental material.

The block randomization method will be used for 1:1:1 randomization to ensure a balanced distribution. Randomization will be attributed by calculating using computer program software.

Researchers responsible for evaluations, application of treatment protocol, and statistical analysis will be blinded to this information. The patient will also be blinded to group allocation. All involved (researchers and patients) in the trial will be instructed not to communicate or comment on which treatment they will receive other than cardiorespiratory rehabilitation. Only at the end of the trial will the identity of the groups and the final results be revealed through patient reports.

### 2.6 Evaluation procedure

(1) Evaluation 1 (baseline), first day of cardiorespiratory rehabilitation
(2) Evaluation 2 at approximately 4.5 weeks of cardiorespiratory rehabilitation (Intermediary)
(3) Evaluation 3 at approximately week 9 of cardiorespiratory rehabilitation (final)
(4) Evaluation 4 follow-up, 2 months following ending of cardiorespiratory rehabilitation.

Data will be stored on the researchers’ computers and in the cloud with shared access among researchers involved in the trial. The evaluation and evolution sheets will be archived within the Laboratory of Sensory-Motor Rehabilitation Engineering, in addition to the digital copy, also stored in the cloud.

### 2.7 Intervention

#### 2.7.1 Schedule

Procedures will be performed twice a week on alternate days for 9 weeks, totaling 18 sessions of therapy, with each session lasting approximately 1 hour.

Patients will be allowed to access the researchers by telephone and email in case of any questions regarding the project or any symptoms.

#### 2.7.2 Cardiorespiratory rehabilitation and photobiomodulation procedures

The treatment session will be divided into following 5 steps:

(1) Step 1: Initial seated rest before exercise, vital signs, and PBM Therapy.

Seated rest before exercise period should be 10 minutes. Concurrent with seated rest, vital signs should be measured followed by the PBM procedure.

Vital signs data such as systemic blood pressure, heart rate, respiratory rate, peripheral oxygen saturation, and BORG will be measured.

Group CCR will rest (seated) without PBM or placebo PBM.

Group CR-PBM will receive transcranial PBM Therapy (wavelength 810 nm laser, beam area on the skin 0.028 cm^2^, laser power 100 mW, irradiance 3.5 W/cm^2^, treatment time 30 seconds per point, energy 3 J per point, and fluence of 107.1 J/cm^2^ (Irradia, Stockholm, Sweden). The location of PBM Therapy will be in the middle cerebral arteries and the anterior cerebral artery (electroencephalogram points F7, F8, and AFz) according to the International 10–20 System of the electroencephalogram.

Group CR-PlaceboPBM will receive PBM Therapy with the device inactive. The therapist will be blinded to active or placebo mode.

After initial seated rest, vital signs data (systemic blood pressure, heart rate, respiratory rate, peripheral oxygen saturation, and BORG) will be repeated.

(2) Step 2: Aerobic training phase: (i) Warm-up, (ii) Aerobic resistance training, and (iii) Cool down.

**Figure 2:**
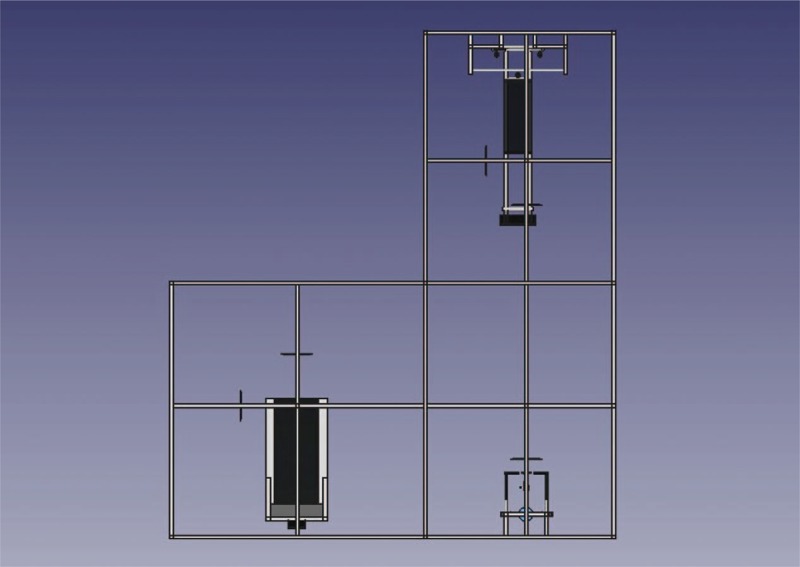
Rehabilitation station and physical activity BrainMov.

These phases will occur on a treadmill (Moviment RT200, São Paulo, Brazil), using support of the BrainMov Rehabilitation and Physical Activity Station (Fig. 2). This is composed of 4 iron pillars, where extensors and elastic cords are added, favoring suspension and stabilization of people with mild to severe impairment of neuromuscular function. This structure allows users to practice dynamic exercises that could not be performed without the help of others. The equipment is safe and thus adapted for rehabilitation and training of people with reduced mobility, providing aerobic training and muscle strengthening.^[25]^

(i) Warm-up: 5 minutes of aerobic heating. The patient will walk at low speed on the treadmill for 5 minutes. Systemic arterial pressure, heart rate, and peripheral oxygen saturation will then be assessed.
(ii) Aerobic resistance training: The individual will walk on the treadmill for 15 minutes, at an intensity according to the patients training heart rate. This will be determined by the Karvonen equation [Training heart rate = % of desired effort x (predicted maximum heart rate - resting heart rate) + resting heart rate], where predicted maximum heart rate is 220-age. The percentage of effort that will be considered in the THR equation will be between 50% and 70%.^[26]^ Monitoring will also be guided via the effort perception BORG scale, maintaining a moderate subjective perception effort (12--14 of BORG scale). At 7 and 15 minutes, systemic blood pressure, heart rate, and peripheral oxygen saturation will again be assessed.^[26]^
(iii) Cool down: 5 minutes. Treadmill speed will be decreased gradually to prevent falling injury. Where patients are walking at the minimum treadmill speed, during aerobic resistance, cool down will occur with the treadmill off, in an upright position. Systemic blood pressure, heart rate, and peripheral oxygen saturation will again be assessed.

After aerobic training, patients will rest (seated) for 5 minutes, following which blood pressure, heart rate, and peripheral oxygen saturation will again be assessed.

(3) Step 3: Respiratory exercises and muscle strengthening phase

Two breathing exercises and 2 muscle strengthening exercises will be performed as follows:

Breathing exercises:

(i) Exercise of diaphragmatic breathing: To promote pulmonary expansion and strengthen of respiratory muscles, the patient will be instructed to breathe in the diaphragmatic pattern, where in the inspiratory phase, the air must be inspired by the nostrils, contracting the diaphragm, so that the abdominal and thoracic region spreads (mainly the abdomen). In the expiratory phase, the air must be exhaled by the mouth, associated with abdominal contraction. This will be performed in a sitting position. Patients will perform 3 sets of 8 repetitions.
(ii) Fractionally inspiratory breathing exercise: In the seated position, patients will be instructed to perform 3 brief and consecutive inspirations, in order to achieve total lung capacity. After a short inspiratory pause, the individual will be instructed to release all the air through the mouth (exhalation) slowly, smoothly, and with half-closed lips. Patients will perform 3 sets of 5 repetitions.

Muscle strengthening exercises:

(i) Exercise of muscular strengthening in closed kinetic chain of hamstrings, femoral quadriceps, gluteus maximus, and gluteus maximus: Crouching movement, holding in the back, and aided by the Rehabilitation and Physical Activity Station BrainMov. Patients will perform 3 sets of 10 repetitions.
(ii) Exercise of muscle strengthening in the open kinetic chain of the iliopsoas and quadriceps femoris groups: Hip and knee flexion movement, facilitating movement of triple flexion during gait. Patients will be able to hold the movement to the back and will be assisted by the BrainMov Rehabilitation and Physical Activity Station to perform the exercise. Patients will perform 3 sets of 10 repetitions.

(4) Step 4: Relaxation phase 5 minutes: Global stretches will be performed for upper limbs and lower limbs, actively associated with slow and gentle breathing.
(5) Step 5: Cardiorespiratory rehabilitation and activities of daily life guidelines: In this stage, the patients participating in the trial and their families will be provided with guidelines to facilitate daily life activities and elucidation about the cardiorespiratory rehabilitation process.

Finally, systemic blood pressure, heart rate, and peripheral oxygen saturation will again be assessed.

#### 2.7.3 Safety measures

During PBM Therapy, patient, therapist, and any observers will wear safety laser goggles with suitable optical density for the wavelength and intensity used.

For hygiene, the laser probe tip will be cleaned up between patient applications. Vital signs and effort perception (by the scale BORG and training heart rate by the Karvonen equation) will be monitored throughout, in order to identify any changes outside normal physiological ranges. If the peripheral oxygen saturation decrease is equal to or less than 88%, the patient will be supplemented with oxygen, adjusted as necessary. An oxygen gas cylinder, nasal catheter and mask, and ready-to-use air humidification system will be available at the site.

To reduce the minimum risk of falls, in the case of dizziness, abrupt changes in vital signs, or motor function, there will always be 2 physiotherapists close to the patient to support. The treadmill will be immediately disconnected by one of the therapists, and as a preventive measure, patients will use the BrainMov Rehabilitation and Physical Activity Station equipment to maintain greater stability and suspension if they lose their balance.

Patients may discontinue treatment at any time if they feel sick or feel any discomfort.

Should a patient present worsening of any symptom caused by the therapy, it will be immediately interrupted, and if necessary, the patient will be rescued.

### 2.8 Outcome measures

(1) Muscle electrical activity (surface electromyograph, 830 Wi-fi, EMG System do Brasil, Brazil): Surface electrodes will be positioned on the rectus femoris and biceps femoris muscles, during the squatting movement: 10 seconds of signal collection (in triplicate);
(2) Bipodal balance during the squat movement: EMG balance evaluation will be performed during the squat movement in the force platform (stabilometric analysis in kgf), 10 seconds of signal collection (in triplicate);
(3) Pulmonary function (Cosmed MicroQuark Spirometer, Brazil): Using the spirometry technique, forced expiratory volume in the first second; Tiffeneau index and maximal voluntary ventilation will be evaluated by respiratory maneuvers. Volume of air exhaled in the first second of the forced vital capacity maneuver will be evaluated.
(4) Respiratory muscle strength (inspiratory and expiratory): Respiratory muscle strength will be evaluated by the analog manovacuometer, 0 ± 300 cmH_2_O, Ger-Ar;
(5) Expiratory peak analysis: force and velocity of airflow from inside the lungs using the Peak Flow Meter, NCS expiratory flow meter.
(6) Thoracic-abdominal expansion and mobility: A common metric tape (1.5 m) positioned at the axillary, xiphoid, and abdominal levels will be used, which will measure inspiratory and expiratory mobility values and be subtracted (inspiration - expiration).
(7) Blood circulation: Assessed with an infrared thermographic camera (ThermaCam FLIR S65HS, Sweden), blood circulation of the lower limbs will be evaluated by temperature difference;
(8) Heart rate variability as an indication of autonomic nervous system responses: assessed by means of a frequency meter (Polar RS800, Finland, California) during treadmill aerobic training for 40 minutes (5 minutes initial seated rest, 30 minutes of exercise, and 5 minutes of final seated rest). R-R intervals will be analyzed;
(9) Blood lactate level: measured by a lactometer with lactate reagent tapes (Accutrend PLUS from Roche). Blood samples will be collected before and after treadmill beginning aerobic training;
(10) 6-minute walk test (Adapted): The 6-minute walk test will be adapted for neurological patients: evaluation of parameters of exercise tolerance on treadmill (Moviment RT200, Brazil) and vital signs responses during and recovery. Patients will be assisted by the BrainMov Rehabilitation and Physical Activity Station to stabilize the trunk and thus stand erect to walk on the treadmill;
(11) Quality of life using the Short Form 36: assessing domains of functional capacity, physical appearance, pain, general health, vitality, social aspect, emotional spectrum, and mental health;
(12) Cognitive functions: using the Mini-Mental State Examination.

### 2.9 Statistical analysis

Data will be stored in Excel (Windows) and in the cloud, organized as mean and standard deviation. Statistical analysis will be carried out within the University of Vale do Paraíba institution. Assumption of normality will be checked, to direct the choice of appropriate statistical test used for intra and intergroup comparisons. Statistical significance will be adopted as *P* < .05. Scientific Data Analysis and Visualization (SciDAVis) will be the software of choice.

## 3 Discussion

New therapies are evolving to improve quality of life for patients affected by diseases of the central nervous system, and there has been a focus on physical conditioning to enable improvement in social activities and activities of daily living. Individuals with neurological impairment have decreased cardiorespiratory fitness, generally perform insufficient recommended physical activity, either through sedentary sequelae or habit.^[12]^ Sedentary individuals are at a higher risk of death and are twice as likely to suffer from cardiovascular disease, compared with physically active persons.^[27]^

Residual motor limitation, as a result of neurological impairment, causes difficulty in muscle recruitment, altered muscle tone, decreased resistance, and high energy expenditure. A sedentary lifestyle also promotes changes in the autonomic system, which directly impacts cardiac tissue functionality, and leads to negative peripheral cardiovascular adaptations, such as reduction of artery diameter and increased vascular resistance. Aerobic training, in addition to potentiating functional recovery, stimulates a neuroprotective effect in the brain, acting on cortical and subcortical regions.^[11–29]^

Aerobic exercise is a part of cardiovascular rehabilitation in these patients, where the main objectives are to reduce cardiovascular risks and to rehabilitate the patient in an integral way, offering support in physical, psychological, social, vocational, and spiritual aspects of quality of life^[26]^ Saunders et al ^[30]^ argue that there is a strong argument that cardiorespiratory training involving walking should be inserted into post-stroke rehabilitation programs, as it stimulates function, cadence, and gait tolerance. Moreover, economic evaluation analysis shows that the effectiveness of cardiovascular rehabilitation brings clinical benefits (years of life earned). When this is divided by the monetary value (cost), this manifests in a reduction in the cost of each additional year of life, in comparison with alternative treatments or absence of treatment.

According to Coote et al,^[16]^ the scarcity of studies investigating medium- and long-term benefits of aerobic training and resistance interventions in individuals with multiple sclerosis is of concern. However, a minimum of 30 minutes of moderate-intensity aerobic exercise and resistance training twice a week is recommended, based on area guidelines. In order to promote positive cardiovascular adaptations and improved respiratory capacity, Van der Scheer et al^[31]^ report that adult individuals with spinal cord injury should perform at least 20 minutes of vigorous intensity aerobic activity and 2-fold strength training exercises per week. Chin et al^[32]^ carried out a 12-week supervised aerobic training program in patients with traumatic brain injury and demonstrated improvements in cardiorespiratory fitness and fatigability. Their study highlighted the importance of performing exercise at the correct intensity for each individual. PBM Therapy, formerly known as low-level laser therapy, is able to trigger a range of physiological effects, through increased cellular metabolic energy production and reduced oxidative stress. These effects occur due to the increased activation of cytochrome C oxidase (the terminal enzyme of the electron transport chain) through light absorption, leading to improved oxygen consumption, cerebral blood flow, and neuronal metabolic capacity.^[33]^

A number of PBM Therapy research studies have investigated attention, memory, anxiety and depression, as well as increased cortical oxygenation. The literature describes PBM Therapy as a safe, noninvasive treatment, with no reported side effects.^[33,34]^

We hypothesize that exposure of neural tissue to PBM Therapy could, through vasodilation and increased cerebral perfusion, also lead to positive effects peripherally. Therefore, we propose that combining the effects of aerobic exercise and PBM Therapy will increase performance in physical activity in patients affected by diseases of the central nervous system.

## Author contributions

Conceptualization: Ana Paula Pinto, Carolina Lobo Guimarães, Gabriela Aparecida da Silveira Souza, Aline Priscila Campos Pereira, Marcele Florêncio das Neves;

Data curation: Ana Paula Pinto, Carolina Lobo Guimarães, Marcele Florêncio das Neves;

Formal analysis: Ana Paula Pinto, Carolina Lobo Guimarães;

Investigation: Ana Paula Pinto, Carolina Lobo Guimarães, Gabriela Aparecida da Silveira Souza, Aline Priscila Campos Pereira;

Methodology: Ana Paula Pinto, Carolina Lobo Guimarães, Gabriela Aparecida da Silveira Souza, Aline Priscila Campos Pereira, Fernanda Pupio Silva Lima, Mário Oliveira Lima, Patrícia Sardinha Leonardo, Rodrigo Alvaro Brandão Lopes-Martins;

Project administration: Ana Paula Pinto, Carolina Lobo Guimarães, Mário Oliveira Lima, Rodrigo Alvaro Brandão Lopes-Martins;

Supervision: Fernanda Pupio Silva Lima, Mário Oliveira Lima, Rodrigo Alvaro Brandão Lopes-Martins

Validation: Ana Paula Pinto, Carolina Lobo Guimarães; Fernanda Pupio Silva Lima, Mário Oliveira Lima, Patrícia Sardinha Leonardo, Rodrigo Alvaro Brandão Lopes-Martins

Visualization: Ana Paula Pinto, Carolina Lobo Guimarães, Gabriela Aparecida da Silveira Souza, Aline Priscila Campos Pereira;

Writing – original draft: Ana Paula Pinto, Carolina Lobo Guimarães;

Writing – review & editing: Ana Paula Pinto, Carolina Lobo Guimarães, Fernanda Pupio Silva Lima, Mário Oliveira Lima, Patrícia Sardinha Leonardo, Rodrigo Alvaro Brandão Lopes-Martins.

**Conceptualization:** Mário Oliveira LIma, Patrícia Sardinha Leonardo, Marcele Florêncio Das Neves, Fernanda Púpio Silva Lima, Rodrigo Alvaro Brandão Lopes Martins.

**Data curation:** Ana Paula Pinto, Carolina Lobo Guimarães, Gabriela Aparecida Silveira Souza, Marcele Florêncio Das Neves.

**Formal analysis:** Carolina Lobo Guimarães, Marcele Florêncio Das Neves, Rodrigo Alvaro Brandão Lopes Martins.

**Investigation:** Ana Paula Pinto, Carolina Lobo Guimarães, Marcele Florêncio Das Neves, Fernanda Púpio Silva Lima, Rodrigo Alvaro Brandão Lopes Martins.

**Methodology:** Ana Paula Pinto, Gabriela Aparecida Silveira Souza.

**Project administration:** Carolina Lobo Guimarães, Gabriela Aparecida Silveira Souza, Patrícia Sardinha Leonardo.

**Supervision:** Fernanda Púpio Silva Lima.

**Validation:** Mário Oliveira LIma.

**Visualization:** Mário Oliveira LIma.

**Writing – original draft:** Mário Oliveira LIma, Ana Paula Pinto, Carolina Lobo Guimarães, Rodrigo Alvaro Brandão Lopes Martins.

**Writing – review & editing:** Mário Oliveira LIma, Patrícia Sardinha Leonardo, Fernanda Púpio Silva Lima, Rodrigo Alvaro Brandão Lopes Martins.

## Supplementary Material

Supplemental Digital Content
