# Supplementary material for: Sensory-motor and cardiorespiratory sensory rehabilitation associated with transcranial photobiomodulation in patients with central nervous system injury: Trial protocol for a single-center, randomized, double-blind, and controlled clinical trial
Source: Medicine (Baltimore). 2019 Jun 21;98(25):e15851. doi: 10.1097/MD.0000000000015851 (PMC6636949; doi:10.1097/MD.0000000000015851)
Supplement: Supplemental Digital Content [file medi-98-e15851-s001.docx]

**Appendices**

**FREE AND EXCLUDED CONSENT TERM**

**Identification info**

**Project Title: SENSORY-MOTOR REHABILITATION AND CARDIORRESPIRATORY ASSOCIATED WITH TRANSCRANIAN LASERTHERAPY IN PATIENTS WITH INJURY IN THE CENTRAL NERVOUS SYSTEM.**

Principal Researcher: Physiotherapist Ana Paula Pinto.

Executive team: Physiotherapists Ana Paula Pinto and Carolina Lobo Guimarães, Prof. Dr. Mário Oliveira Lima and Prof. Dr. Rodrigo Alvaro Brandão Lopes Martins.

Institution to which the Researcher in charge belongs: University of Vale do Paraíba, Research and Development Institute.

Telephones for contact - Reception of the physiotherapy clinic University of Vale do Paraíba: (12) 3947-1086

Researcher's cell phone: (12) 98158-5874 / (12) 98826-2896

Ethics and Research Committee of University of Vale do Paraíba: (12) 3947-1111

Name: __________________________________________________

Age: _______ R.G. ____________________ CPF: ________________________

Adress: __________________________________________________________

Contact Phone: ________________________________________________

You ________________________________________ is being invited to take part in the research project entitled "SENSORY-MOTOR REHABILITATION AND CARDIORRESPIRATORY ASSOCIATED WITH TRANSCRANIAN LASERTHERAPY IN PATIENTS WITH INJURY IN THE CENTRAL NERVOSO SYSTEM", by the researcher Ana Paula Pinto.

This research is justified since other studies have already shown benefits for people who perform aerobic activities. Therefore, the main objective of this work will be to evaluate the effect of laser therapy and aerobic exercise on muscle activation, balance, cardiorespiratory fitness and quality of life through a rehabilitation and training program for people with reduced mobility.

In addition to aerobic activities (cardiorespiratory rehabilitation), low-intensity laser therapy, a non-invasive, non-painful, non-heated and safe therapy will also be offered. This therapy consists of the application of a light, generated by an appliance, and when in contact with our body, generates different benefits, depending on the application site, but in general, this treatment reduces inflammation, pain, improves healing, muscle fatigue, among others. In this trial the light will be applied in three points: in the center of the forehead and in the region of the temples (region above the ears, on the right and left side), since other studies demonstrated that when the laser was applied in these regions, the memory improved, circulation and decreases symptoms of depression and anxiety.

The survey will have 3 groups. Group 1 (G1) patients will only participate in cardiorespiratory rehabilitation; in group 2 (G2) in addition to cardiorespiratory rehabilitation, will also receive the treatment of low intensity laser therapy, which will be applied before the beginning of exercise; and group 3 (G3), patients will receive cardiorespiratory rehabilitation, and simulation of laser therapy treatment (placebo), which the laser device will be switched off. The patients will be drawn in one of the groups, and in case you are drawn to the G2 or G3, by security measures, during the application of the laser, you and other people present on the premises will be equipped with safety glasses suitable for the light used, and the tip of the apparatus where the light comes out, will be covered by a protective plastic film.

All patients, after completing the trial, may receive low-intensity laser treatment. So, if you are randomized during the trial in a group that does not receive laser therapy (G1 and G3), if you are interested, you can receive this treatment later.

You will be submitted to 4 evaluations: Before starting the rehabilitation, after the 9th session, at the end of the rehabilitation and after 2 months of the end of the treatment. Vital signs, lung tests, fatigue (blood lactate), exercise tolerance will be collected, and two questionnaires will be applied: one for cognitive evaluation and the other for quality of life. The access to the answers of the questionnaires will be confidential to the researchers of this research, respecting their privacy. The collection of blood lactate by the blood droplet will be done with a small perforation at the tip of one hand, performed with the aid of a disposable needle, by the responsible researcher, at the Laboratory of Sensory-Motor Rehabilitation Engineering (UNIVAP). All hygienic and safety measures will be used throughout the test, including the use of disposable gloves by the researcher. These tests do not cause pain and eventually only slight tiredness can be felt.

The rehabilitation process will be approximately 2 months of treatment, 2x per week, with about 1 hour each session, and at least 24 hours apart. This trial will be carried out at the Center of Supervised Practices and in the Laboratory of Sensory Motor Rehabilitation Engineering at the University of Vale do Paraíba, located at Av. Shishima Hifumi, 2911, Urbanova, blocks 7 and 9.

During rehabilitation, mild fatigue, abnormal changes in vital signs, decreased oxygenation and minimal risk of falls may occur. However, steps will be taken to minimize these risks and make therapy safer, and in case of any intercurrence, those in charge of the trial will provide full assistance to the patients. Thus, the patient may interrupt the sessions or withdraw from this research at any time, without prejudice to follow-up or suffer any sanctions or constraints.

If necessary, you will be assisted by the SAMU ambulance of the city of São José dos Campos and sent to the municipal hospital Dr. José de Carvalho Florence of São José dos Campos, namely Hospital of the Industrial Village, where you will receive medical assistance by Unified Health System.

You will be entitled to compensation at any stage of the research if there is any immediate or late damage (with or without a forecast) resulting from your participation in the research.

I affirm that I have accepted to participate of my own free will, without receiving any financial incentive or have any liens and with the exclusive purpose of collaborating for the success of the research.

I was also informed that my information will be subject to the ethical standards for research involving human beings, of the National Commission for Research Ethics of the National Health Council of the Ministry of Health. My collaboration will be done in a anonymous, by means of data collection of the evaluations and reassessments. The access to the answers of the questionnaires and analysis of the other data collected will be done only by the researchers, respecting the confidentiality and privacy of their data. I certify receipt of a signed copy of this Free and Informed Consent Form, as recommended by the National Commission for Research Ethics. I have also been informed that I can contact / consult at any time I deem necessary the responsible researcher Ana Paula Pinto and all the executing team: Carolina Lobo Guimarães, Prof. Dr. Mário Oliveira Lima and Prof. Dr. Rodrigo Alvaro Brandão Lopes Martins for assistance and / or to answer any questions about the procedures, risks, benefits and other matters related to the research or individual treatment through the telephones nº 3947-1086 or e-mail apaula@outlook.com .br / caroll.guimaraes @ yahoo.com.br or in person at Av. Shishima Hifumi, 2911, Urbanova, block 07- from Monday to Friday, from 8h to 17h. This research protocol was approved by the Research Ethics Committee where it is jointly responsible for ensuring and ensuring the rights of the research patient - whose contact data are: Research Ethics Committee of the University of Vale do Paraíba , by telephone ( 12) 3947-1111, or in person at Av. Shishima Hifumi, 2911, Urbanova, block 11 - Research and Development Institute II, room 19, from Monday to Friday, from 8:00 a.m. to 12:00 p.m. and from 1:00 p.m. to 5:00 p.m.

I, ___________________________________________________________, RG nº _____________________ was informed and agree to participate as a patient in the research project described above.

São José dos Campos, _____ de ____________ de _______

____________________________ ______________________________

Name and Signature Name and signature of person responsible for obtain consent

___________________________ ___________________________

Witness Witness
